# Comment on “The risk of malignancy in patients with IgG4-related disease: a systematic review and meta-analysis” by Yu et al.

**DOI:** 10.1186/s13075-022-02789-8

**Published:** 2022-05-24

**Authors:** Hann-Ziong Yueh, James Cheng-Chung Wei, Liyun Zhang

**Affiliations:** 1 Department of Education, Chung Shan Medical University Hospital, Taichung, Taiwan; 2 Department of Allergy, Immunology & Rheumatology, Chung Shan Medical University Hospital, Taichung, Taiwan; 3 Institute of Medicine, Chung Shan Medical University, Taichung, Taiwan; 4 Graduate Institute of Integrated Medicine, China Medical University, Taichung, Taiwan; 5 Department of Rheumatology, Third Hospital of Shanxi Medical University, Shanxi Bethune Hospital, Shanxi Academy of Medical Sciences, Tongji Shanxi Hospital, Taiyuan, Shanxi China

**Keywords:** IgG4-RD, Malignancy, Pseudotumors, Meta-analysis

## Abstract

With great interest, we have read the recent article “The risk of malignancy in patients with IgG4-related disease: a systematic review and meta-analysis” by Yu et al. While we have a great appreciation for the work conducted by the authors there are some methodological issues need to be considered. First, the period of articles included in the study, almost before 2013, implied that most follow-up days in these articles were earlier than the established date of a unified definition of IgG4-RD, 2011. Thus, it may lead to misclassification bias in the study. Second, IgG4-RD is a fibrous-inflammatory process that often involves multiple organs; however, malignant tumors related to IgG4-RD proposed in the study were only confined to four diseases. Therefore, we suggest adding subgroup analysis for more malignancies depending on the prevalence of IgG4-RD involved organs to ensure better clinical practice. Third, the causation between IgG4-RD and malignancy remains obscure currently. The time course for development in different malignancies varies significantly so that we cannot infer that malignancies discovered after IgG4-RD are directly relevant. With problems mentioned above, we recommend solutions to make this article more convincing.

## Main text

Dear Editor,

With great interest, we have read the recent article “The risk of malignancy in patients with IgG4-related disease: a systematic review and meta-analysis” by Yu et al. [1]. published in Arthritis Research and Therapy. While we have a great appreciation for the work conducted by the authors, there are some methodological issues that need to be considered.

First, the calendar period of 10 articles included in the study, almost before 2013, implied that most follow-up days in these articles were earlier than the established date of unified definition of IgG4-RD, 2011. Before 2011, several diseases such as Mikulicz disease, Kuttner’s tumor, and Riedel thyroiditis were not categorized into comprehensive diagnostic criteria for IgG4-RD published by the Japanese IgG4 team, organized by the Ministry of Health, Labor and Welfare of Japan. Therefore, misclassification bias, which occurs when an individual is assigned to a different category than the one to which they should be assigned, may exist in the study. These cases before 2011 may be underdiagnosed due to the lack of complete reports of serum IgG or histopathological findings. Besides, the organ-specific criteria have already included IgG4-related autoimmune pancreatitis, IgG4-related Mikulicz’s disease, and kidney disease, but they are not suitable for the diagnosis of patients with involvement of other organs [2]. With greater awareness and emphasis from clinical physicians, the definition of IgG4-RD has been revised annually in recent years [3]. Therefore, we recommend subgroup analysis before and after 2011 (Lee et al. [4]) and collect more recent articles after 2011 to make this meta-analysis more convincing.

Second, IgG4-RD is a fibrous-inflammatory process that often involves multiple organs, either synchronously or metachronously, including cervical lymph nodes, lung, liver, bile duct, gastrointestinal tract, central nervous system, thyroid, pancreas, kidney, prostate, arteries, lymph nodes, skin, breast, lacrimal glands and salivary gland [5]. Although organ distribution varies among studies, the pancreas, bile ducts, and salivary glands are the most frequently involved organs [2, 6]. However, malignant tumors related to IgG4-RD proposed in the study were only confined to four diseases (pancreatic cancer, lymphoma, gastric cancer, and lung cancer). Therefore, we suggest adding subgroup analysis for more malignancies depending on the prevalence of IgG4-RD involved organs to ensure better clinical practice.

Third, the causation between IgG4-RD and malignancy remains obscure. IgG4-RD has ambiguous presentations with pseudotumors which can be disseminated in the affected organs, mimicking metastases. For IgG4-RD, PET/CT is utilized to detect the disease activity and determine the extent of organ involvement [7]. It is more likely to have the accidental discovery of neoplasms in other organs that cause so-called a surveillance bias. Besides, the time course for development in different malignancies varies significantly so that we cannot infer that malignancies discovered after IgG4-RD are directly relevant. It is perhaps the influence of time sequence of diagnosis. Therefore, it is not suitable for meta-analysis up till the present moment unless the blurred boundary between these two diseases is eliminated by clear clarification.

## Data Availability

Not applicable.
